# Eastern Ohio Dental Association

**Published:** 1872-10

**Authors:** 


### Eastern Ohio Dental Association.

The third semi-annual meeting of the Eastern Ohio Dental Association was held at the office of Dr. J. C. Whinery, in Salem, Ohio, on Tuesday, August 27, 1872.

The Society was called to order by the President, Dr. Whinery. Dentists present: L. C. Hole, J. C. Whinery, G. A. Richards, T. A. Lewis, Salem; A. J. Douds, Canton; J. W. Lyder, C. P. Coffee, Alliance; W. P. Rice, Mt. Union; J. M. Porter, Massillon; D. B. McLain, New Lisbon; B. F. Gibbons, Youngstown; D. Gibbons, Warren; J. T. Barclay, Columbiana. Minutes of last meeting read and approved. The society then elected the following officers for the coming year: President, A. J. Douds; Vice President, L. C. Hole; Secretary, J. M. Porter; Treasurer, J. C. Whinery; Examining Committee, J. W. Lyder, J. C. Whinery, J. M. Porter.

The retiring President, Dr. Whinery, on leaving the chair, made a brief speech, thanking the members for their co-operation, and encouraging them to raise the standard of dentistry in Eastern Ohio, by doing away with all feeling of jealousy, and by cultivating a more generous and social feeling toward each other. This being a local society, he urged the members present to remember that their individual success, depended upon the standing of the profession in their respective locations. The doctor in his remarks was brief and practical, as he *always* is. Dr. Douds, the President elect, then assumed his duties as President. On motion of Dr. Whinery, the members of the medical and dental professions present were invited to participate in the discussions. The first subject discussed was, "The treatment and filling of diseased teeth and roots;" discussed by Drs. Porter and Lyder. The subject of "Dental Ethics" was then discussed by Drs. Whinery, Porter, D. Gibbons, B. F. Gibbons, Lewis, and Lyder. The society then adjourned to one o'clock P. M. At the appointed time the society met, and a short clinic was held on "Excavating," each member present giving his views. An essay on "Diagnosis" was then read by J. M. Porter, followed by an essay on "The sympathetic affections arising from dental irritation," by Dr. Douds. Both essays were accepted by the society. The subject of "Neuralgia" was then freely discussed, and a number of interesting cases reported. J. M. Porter was then called upon for a fifteen-minute speech on "Specialties in Dentistry," being an advocate of this system of practice.

"How to preserve children's teeth," and "Artistic Dentistry," were selected as the subjects for discussion at the next meeting. Drs. Whinery and Rice were appointed essayists for next meeting. The society then adjourned, to meet in Alliance on the last Tuesday in February, 1873.

